# 3-Dimensional mesothelioma spheroids provide closer to natural pathophysiological tumor microenvironment for drug response studies

**DOI:** 10.3389/fonc.2022.973576

**Published:** 2022-08-26

**Authors:** Huaikai Shi, Emma M. Rath, Ruby C. Y. Lin, Kadir Harun Sarun, Candice Julie Clarke, Brian C. McCaughan, Helen Ke, Anthony Linton, Kenneth Lee, Sonja Klebe, Joanneke Maitz, Kedong Song, Yiwei Wang, Steven Kao, Yuen Yee Cheng

**Affiliations:** ^1^ Asbestos Diseases Research Institute, Concord, Sydney, NSW, Australia; ^2^ Giannoulatou Laboratory, Victor Chang Cardiac Research Institute, Sydney, NSW, Australia; ^3^ Centre for Infectious Diseases and Microbiology, The Westmead Institute for Medical Research, Sydney, NSW, Australia; ^4^ Sydney Medical School, University of Sydney, Sydney, NSW, Australia; ^5^ School of Medical Sciences, University of New South Wales, Sydney, NSW, Australia; ^6^ Sydney Cardiothoracic Surgeons, Royal Prince Alfred Hospital (RPA) Medical Centre, Sydney, NSW, Australia; ^7^ Concord Repatriation General Hospital, Sydney, NSW, Australia; ^8^ Pathology, Flinders Health and Medical Research Institute, Flinders University, Bedford Park, SA, Australia; ^9^ The ANAZC Research Institute, Sydney, NSW, Australia; ^10^ State Key Laboratory of Fine Chemicals, Dalian R&D Center for Stem Cell and Tissue Engineering, Dalian University of Technology, Dalian, China; ^11^ Jiangsu Provincial Engineering Research Centre of Traditional Chinese Medicine (TCM) External Medication Development and Application, Nanjing University of Chinese Medicine, Nanjing, China; ^12^ Institute for Biomedical Materials & Devices, Faculty of Science, The University of Technology Sydney, NSW, Australia

**Keywords:** 3D spheroids, mesothelioma, tumor microenvironment, decellularized lung scaffold, microRNA expression, drug response and resistance

## Abstract

Traditional studies using cancer cell lines are often performed on a two-dimensional (2D) cell culture model with a low success rate of translating to Phase I or Phase II clinical studies. In comparison, with the advent of developments three-dimensional (3D) cell culture has been championed as the latest cellular model system that better mimics *in vivo* conditions and pathological conditions such as cancer. In comparison to biospecimens taken from *in vivo* tissue, the details of gene expression of 3D culture models are largely undefined, especially in mesothelioma – an aggressive cancer with very limited effective treatment options. In this study, we examined the veracity of the 3D mesothelioma cell culture model to study cell-to-cell interaction, gene expression and drug response from 3D cell culture, and compared them to 2D cell and tumor samples. We confirmed *via* SEM analysis that 3D cells grown using the spheroid methods expressed highly interconnected cell-to-cell junctions. The 3D spheroids were revealed to be an improved mini-tumor model as indicated by the TEM visualization of cell junctions and microvilli, features not seen in the 2D models. Growing 3D cell models using decellularized lung scaffold provided a platform for cell growth and infiltration for all cell types including primary cell lines. The most time-effective method was growing cells in spheroids using low-adhesive U-bottom plates. However, not every cell type grew into a 3D model using the the other methods of hanging drop or poly-HEMA. Cells grown in 3D showed more resistance to chemotherapeutic drugs, exhibiting reduced apoptosis. 3D cells stained with H&E showed cell-to-cell interactions and internal architecture that better represent that of *in vivo* patient tumors when compared to 2D cells. IHC staining revealed increased protein expression in 3D spheroids compared to 2D culture. Lastly, cells grown in 3D showed very different microRNA expression when compared to that of 2D counterparts. In conclusion, 3D cell models, regardless of which method is used. Showed a more realistic tumor microenvironment for architecture, gene expression and drug response, when compared to 2D cell models, and thus are superior preclinical cancer models.

## Introduction

In the past two decades, cancer research has revealed that the tumor microenvironment (TME) plays a critical role in cancer development and progression. There are two major components of the TME: the cellular and non-cellular components. The cellular components include cancer-associated fibroblasts, tumor infiltrating mesenchymal stem cells, tumor infiltrating lymphocytes and endothelial cells that interact with tumor cells. The role of these cells in tumor cell proliferation, migration and therapeutic resistance has been widely investigated (1). The non-cellular components of the TME, including the extracellular matrix (ECM), growth factors, cytokines and chemokines, also play a significant role in cancer progression by presenting cues that affect fundamental aspects of tumor cell biology (2).

Cancer biology and cancer drug screening studies still heavily use conventional two-dimensional (2D) cell culture systems for research. The time-honored 2D cell culture model of growing a monolayer of cells on a plastic plate has proven to be valuable but has significant limitations in its ability to model the TME, cell polarity and signaling, ECM production, gene expression, architectural features, and response to oxygen, which are all important factors that will affect cancer biology and drug screening results. As a consequence, initial anti-cancer drug tests were often found to be inefficient due to the poor correlation between 2D cell culture models and human pathophysiology (3–5). Animal models are widely accepted and used in cancer research to mimic the TME *in vivo*, but there are limitations associated with their use. Animal model-based experiments are costly, time-consuming, labor intensive and are less amenable to large-scale screening (6, 7). In addition, responsible ethics practices encourage the use of alternatives to animal models where possible. Therefore, the development of next-generation three-dimensional (3D) cell culture models has attracted growing interest in cancer research. 3D cell culture microenvironments more closely represent the *in vivo* environment and are a superior model to the conventional 2D system for studying cell-to-cell and cell-to-matrix interactions, nutrient and oxygen gradients, and overall cellular architecture. The 3D model also allows for biological responses to the cell-to-matrix interactions and more closely represents the formation and progression of cancer (8, 9). Therefore, we propose using a novel 3D cell culture model based on decellularized porcine lung cubes to evaluate its efficacy in mesothelioma and lung cancer drug screening.

3D culture systems offer the unique opportunity to culture cancer cells alone or with various cell types in a biologically relevant manner, encouraging cell-to-cell and cell-to-matrix interactions that closely mimic the native TME (10). Previous studies have shown that metabolic, stress response, structural, signal transduction and cellular transport proteins are expressed at elevated levels in spheroids compared to 2D-culture cells (11, 12). The most commonly used 3D culture models of cancer include: a) tumor tissue explant; b) “tumor on a chip”, and c) multicellular tumor spheroids (MCT). “Tumor tissue explant” is one of the earliest 3D models of cancer that involves culturing excised human tumors in tissue culture plates (13). This model has primarily been used for *in vitro* testing of drug efficacy, however, its use in drug screening and cancer research is limited by its low reproducibility owing to the heterogeneity of donor tissue samples. “Tumor on a chip” is a bioengineered biomimetic model that places a tumor functional unit on a microfluidic device, allowing co-culture of tumor cells with other cell types, with microfluidic channels mimicking the vasculature. This model provides new avenues for genomic and drug screening (14). MCT’s are constructed from tumor cells alone, or in combination with other cell types, and are the most characterized organotypic model of cancer. Large MCT’s (>500µm in diameter) have been demonstrated to provide physiochemical gradients similar to micrometastases and avascular tumors and are between 0.5-1 mm^3^ in size due to limited diffusion of oxygen, nutrients, metabolic waste and soluble factors. Lower oxygen levels (hypoxia) in MCT’s were shown to trigger changes in gene expression, promoting aerobic glycolysis and lactic acid production, thus lowering the pH of the inner layer of cells. A large number of cancer cells have been cultured to construct MCT models, including lung cancer cell lines H1437, H356, H2170, A549, Chago K1, H23 and H1703 (1). These MCT models have been demonstrated to possess features that are more suitable for high-throughput screening assays (15).

The poly HEMA 3D model is an MCT that can be generated easily by the liquid overlay technique that prevents matrix deposition. Tumor cells are placed on a plastic tissue culture plate covered with a thin layer of the inert substrate of poly HEMA that is allowed to dry before the addition of medium and cells, allowing cancer cells to grow without adhering, and promoting cell aggregation and compaction. The poly HEMA 3D model has previously been used to examine immunotoxin therapy of human mesothelioma *in vitro* (16). This 3D model is a simple and more accurate representation of *in vivo* tumors and is a model that can be used for further investigations of the effects of the microenvironment on drug penetration and tumor cell death.

3D cells grown in synthetic scaffolds reveal some limitations of inferior cell adhesion due to the defectiveness of natural components. Therefore, natural scaffolds and acellular natural matrices represent a potential platform for tumor tissue engineering based on their biomechanical characteristics and tissue-specific ECM composition. Currently, little information is available on the application of tissue/organ-derived matrix for the development of 3D tumor models. Previous reports have demonstrated that acellular tumor extracellular matrices, produced from xenotransplantation, are a promising 3D model with the ideal spatial arrangement, biomechanical properties and biocompatibility (17). Fecher et al. recapitulated important characteristics of lung tumors and their microenvironment by culturing A549/HCC827 cells on decellularized rat lungs. Furthermore, they demonstrated that the gene expression pattern of cells on the lung scaffold revealed a concordance similar to tumors of patients with a poor prognosis when compared to a Matrigel-based model (18). Therefore, this organotypic tumor model represents a promising system for the reliable analysis of tumor biology and drug testing. In this study, we examine in real-time 3D mesothelioma spheroids in the context of drug response and gene expression and compare them to their 2D counterparts.

## Materials and methods

### Cell lines and cell culture

Three MPM cell lines (H28, H226 and MSTO) and the immortalised mesothelial cell line MeT-5A were obtained from American Type Culture Collection (ATCC, Manassas, VA, USA). The primary mesothelioma cell line MM05 (19) was generated at the University of Queensland Thoracic Research Centre (The Prince Charles Hospital, Brisbane). Primary mesothelioma cells 2175, 1187, and 1157 were established by the ADRI laboratory. Cells were cultured at 5% CO_2,_ 37°C and 95% humidity in RPMI 1640 with 10% fetal bovine serum. All media and FBS were from Life Technologies (Carlsbad, CA, USA). The primary cell used in this project in covered under ETH00873 2022.

### 3D tumor spheroids

3D tumor spheroids were grown by seeding 10,000 cells per well of a 96-well round-bottom suspension culture plate and spinning down at 800 rpm at 5 mins room temperature, cells were cultured as above. Most MPM cells form 3D spheroids at 24 hours post-plating. H226 and MeT-5a normally take 3 days to form 3D spheroids.

### 3D tumor microenvironment with decellularized porcine lung scaffold

Fresh porcine lungs were obtained from 6-month-old pigs. The porcine lungs were washed with saline and partially thawed prior to sections into small cubes at approximately 8-12cm^3^. All segments were checked microscopically without large bronchi. For cell removal, lung segments were immersed in 1% sodium dodecylsulphate (SDS) for 24h and then 0.5% TritonX-100 for 12h. Post decellularization, all segments were washed with PBS and deionized water for 60min followed by freeze-drying to generate porous lung scaffolds. Post freeze-drying, the Lung scaffolds were cross-linked with 50 Mm 1-Ethyl-3-(3-dimethylaminopropyl) carbodiimide (EDC)/N-hydroxy-succinamide (NHS) in morpholine ethane sulfonate (MES) buffer solution for 6 h following secondary freeze-drying.

### Scanning electron microscopy (SEM)

After fixation, dehydrating and drying, cells grown in 3D were mounted on aluminium sample stubs and sputter-coated with platinum using an auto coater at 45nm (JFC-1600 Auto Fine Coater, JEOL Ltd., Tokyo, Japan) prior to examination by scanning electron microscopy (SEM) (JEOL JSM-6380, JEOL Ltd., Tokyo, Japan) at a voltage of 15kV.

### Transmission electron microscopy (TEM)

Cells grown in 3D were fixed in 10% formalin. The specimen was placed in 0.1M sodium cacodylate buffer, post-fixed in 2% osmium tetroxide and treated with 0.5% uranyl acetate. The specimen was dehydrated through a graded series of ethanol, placed in acetone, infiltrated in 50:50 acetone: Spurr’s resin, then embedded in pure Spurr’s resin and polymerized overnight at 70°C. Ultrathin sections of 90 nm were stained with uranyl acetate and lead citrate and examined using a Fei Tecnai Spirit Biotwin electron microscope. Images were obtained with an Olympus-SIS veleta digital camera.

### Drug cytotoxic assay *via* proliferation assay

The rate of *in vitro* cell proliferation was assessed by quantifying increases in DNA measured by the AlamarBlue assay (Rath et al., 2018) in MPM cells grown in 96-well plates (2D) and 96-well round-bottom non-adhesive plates (3D) which were treated with cisplatin (0 to 200μM, in 2-fold dilutions) and gemcitabine (0 to 200 nM in 2-fold dilutions) for 72 hours. To quantify remaining viable cells, Alamar Blue, 20 μl (50 mL PBS containing also Sigma reagents 0.075 g Resazurin, 0.0125 g Methylene Blue, 0.1655 g Potassium hexacyanoferrate (III), 0.211 g Potassium hexacyanoferrate (II) trihydrate, filter-sterilised, and stored at 4°C in the dark), was added and incubated for 2 to 4 hr at 37°C. Fluorescence intensity at 590 ± 10 nm with 544 nm excitation was measured as a percentage of intensity of control cells, using a FLUOstar Optima (BMG LabTech, Ortenberg, Germany).

MPM cells were transfected with a candidate microRNA mimic or control in 96-well plates (2D) and 96-well round-bottom non-adhesive plate (3D). The reintroduction of candidate microRNAs was performed using microRNA mimics. All microRNA mimics were obtained from Shanghai GenePharma. MPM cells were reverse transfected as previously described (20). Briefly, 10,000 cells were reverse transfected with 1 nM of microRNA mimic or control using Lipofectamine RNAiMAX (Life Technologies) at 0.1 µL per well for both 2D and 3D. Cisplatin and Gemcitabine were treated at 24 hrs post-transfected as above and harvested at 72 hrs post drug treatment following cell viability analysis as above. Each experiment was performed in triplicate.

### Apoptosis assay

The Tali Image-Based Cytometer (Thermo Fisher Scientific) was used to measure levels of apoptosis, necrosis, and death in transfected cells after treatment with cisplatin and gemcitabine, using the Tali Apoptosis Kit (Thermo Fisher Scientific). 24 h following transfection with miRNA mimics or controls in 6-well plates, drugs were added to cells at a dose between the IC_50_ values of parental and resistant lines for each respective agent and varied from 50 nmol/L to 3 µmol/L. Following 48 h of drug treatment, cells were resuspended in 100 μL of apoptosis buffer with 5 μL of Annexin V and incubated for 20 min in the dark at room temperature. Samples were then centrifuged, and cell pellets were resuspended in 100 µL of apoptosis buffer containing 1 µL propidium iodide (PI). After 5 min in the dark at room temperature, cells were analysed on the Tali Image-Based Cytometer. Cells were considered to be apoptotic when stained with annexin, dead when stained with PI and late apoptotic/necrotic when stained with both dyes. Each sample was measured across 18-fields of view where total cell numbers for each type of fluorescence were determined with digital image-based counting and fluorescence detection algorithms.

### Immunohistochemistry (IHC)

MPM cell lines were cultured into a 3D spheroid model. Cells were spun down and embedded into cell blocks that were further processed into paraffin blocks. MPM tissue blocks and cell blocks were sectioned at 0.4 *μ*m thickness, deparaffinised, and rehydrated in graded concentrations of xylene and ethanol. Antigen retrieval and immunohistochemical staining were performed on an automated Leica Bond III (Leica Microsystems, Melbourne, Australia) as previously described (21). IHC stained sections were imaged with an Axio Imager.M2 (ZEISS).

### RNA isolation

MPM cells harvested from 2D and 3D cultures were used for total RNA isolation using Trizol reagent (Life Technologies). Isolated RNA was cleaned up by washing with twice 70% ethanol for subsequent analysis.

### MicroRNA profiling

To investigate the differences in the underlying molecular event between 2D and 3D, microRNA profiling was carried out using TaqMan Array Human microRNA A+B Cards Set v 3.0 as previously described (16). Undetermined (Cq=40) data points were removed. The geometric mean of house-keeping genes (HKG), i.e., RNU44, RNU48 and U6snRNA was used to normalize the data. 2D and 3D datasets were analysed separately i.e., 2^-(CqmiR-CqHKG), log 2, and ANOVA analysis was carried out. MPM cell lines (H28, H226, MSTO, MM05) were compared to MeT-5A respectively for 2D and 3D datasets. To note, epithelioid are H28 and H226, and biphasic is MM05 and MSTO. P< 0.05, FDR (false discovery rate based on Benjamini-Hochberg *post hoc* adjustment) was the cut-off.

Due to the nature of microRNAs in dictating up- or down-regulation of specific regulatory pathways, candidate microRNAs were analysed further using miRDB (v5, August 2014 based on miRbase v21) and TargetScan (v7.0, August 2015) for their gene targets.

Pathway enrichment analysis based on KEGG (https://www.genome.jp/kegg/) on the gene targets from miRNA profiling was carried out (**Supplementary Table 3**). Gene enrichment analysis of miRDB gene targets (of the 17 significant differentially expressed microRNAs 3D vs 2D) is based on DAVID gene ID [(https://david.ncifcrf.gov/) using Fisher exact statistics, thresholds by default, Max.Prob.<=0.1 and Min.Count >=2] (**Supplementary Table 2** shows a list of candidate miRNAs, **Supplementary 2** shows DAVID gene enrichment clusters). These analyses were carried out to cross-examine the relationship between microRNA-gene-function outlined in this study. Here, P value is a modified Fisher Exact P value and enrichment (EASE Score) means the smaller, the more enriched.

### Reverse transcription and quantitative real-time PCR (RT-qPCR) and digital PCR

Total RNA extracted from both 2D and 3D cell models were reverse transcribed to cDNA using TaqMan^®^ MicroRNA Reverse Transcription Kit (Life Technologies) following the manufacturer’s protocol. The expression of each candidate microRNA was determined by quantitative real-time PCR using the KAPA PROBE FAST qPCR Kits and Vii7 QPCR System (Life Technologies),. The same cDNA was used for digital PCR analysis using ddPCR™ Supermix for Probes (No dUTP) (BioRad) and BioRad QX200 system. RNU6B was used as a reference gene. microRNA expression levels from qPCR were determined using the 2^-ΔΔCq^ method (22) with normalisation to RNU6B. microRNA expression levels from digital PCR were present as copy numbers per reaction.

### Statistical analysis

MPM cell responses to treatment were modelled using a sigmoid function (23):


$$y=A+(B-A)*\frac{1}{\left(1+\exp\left(\frac{\left(xmid-x\right)}{scale}\right)\right)}$$

where *y* is cell proliferation, *A* is the left asymptote (MPM cell response at drug treatment concentration of 0), *B* is the right asymptote (MPM cell response of percentage of cells that have died at highest drug treatment concentration), *xmid* is the transition point (IC50), *scale* is an x-axis scale parameter impacting slope of the transition, and *x* is log10 of the drug treatment concentration (thus rendering the curve symmetrical and suitable for modeling using log-likelihood). The best fitting parameters for a given model were determined by the maximum log likelihood method, using the Optimax package (24) in R (25).

ANOVA analysis was carried out for microRNA profiling, and pathway and gene analyses with p value<0.05 designated to be significant. *Post-hoc* adjustment using Benjamini-Hochberg was utilized where appropriate.

## Results

### Mesothelioma cells grown in spheroids presented better structure characteristic when compared to 2D counterpart

**Figure 1:**
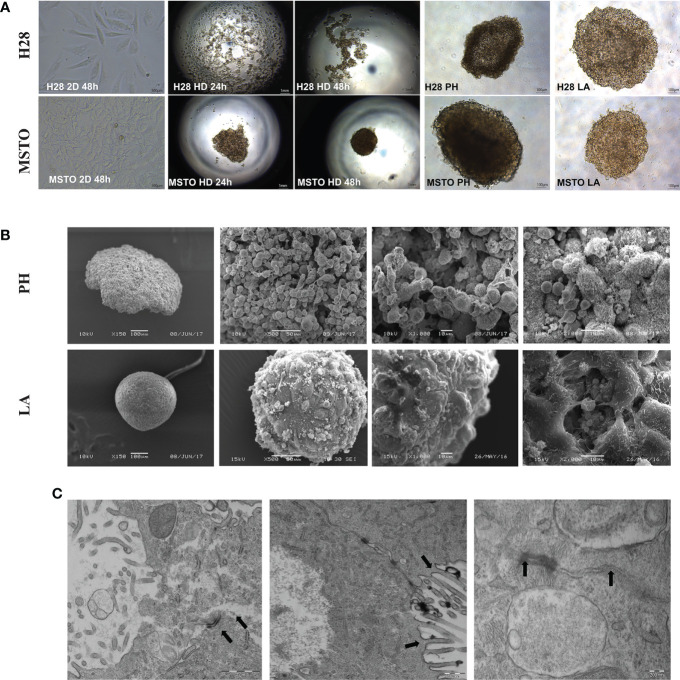
Cancer cells are grown in 2D and 3D conditions. **(A)** H28 and MSTO tumor spheroids, hanging drop (HD), Poly-Hema (PH) and low adhesive plate (LA) were prepared in 3D and visualized using an inverted light microscope 24 and 48 h after 3D spheroid formation. **(B)** SEM was used to study the structure difference of cells grown in 3D using PH (top) and LA (bottom) methods. **(C)** TEM scanning revealed tight cell-to-cell junction and microvilli, indicated by black arrows in the LA spheroids. Scar bar = 200nM.

Firstly, we investigated the arrangement of tumor cells growth in 3D culture compared to 2D. Poly-HEMA (PH) and low adhesive (LA) methods cultured mesothelioma cells (H28 and MSTO) formed uniform spheroid after 24 hours. Cells cultured by hanging drop (HD) method were arranged into spheroids after 48 hours for the MSTO cell line but failed to do so for the H28 cell line (**Figure 1A**). SEM revealed a multi-layer cell structure with a rough surface in poly-HEMA cultured spheroids. In comparison, spheroids grown by low adhesive methods formed a tight structure with a smooth surface (**Figure 1B**). We further investigated the low adhesive model using TEM scanning. TEM scanning showed a clear tight junction between cells and the unique microvilli structure present in the mesothelioma cells (**Figure 1C**).

### Mesothelioma cells grow in 3D demonstrated increased drug resistance with reduced apoptosis when compared to their 2D counterparts

**Figure 2:**
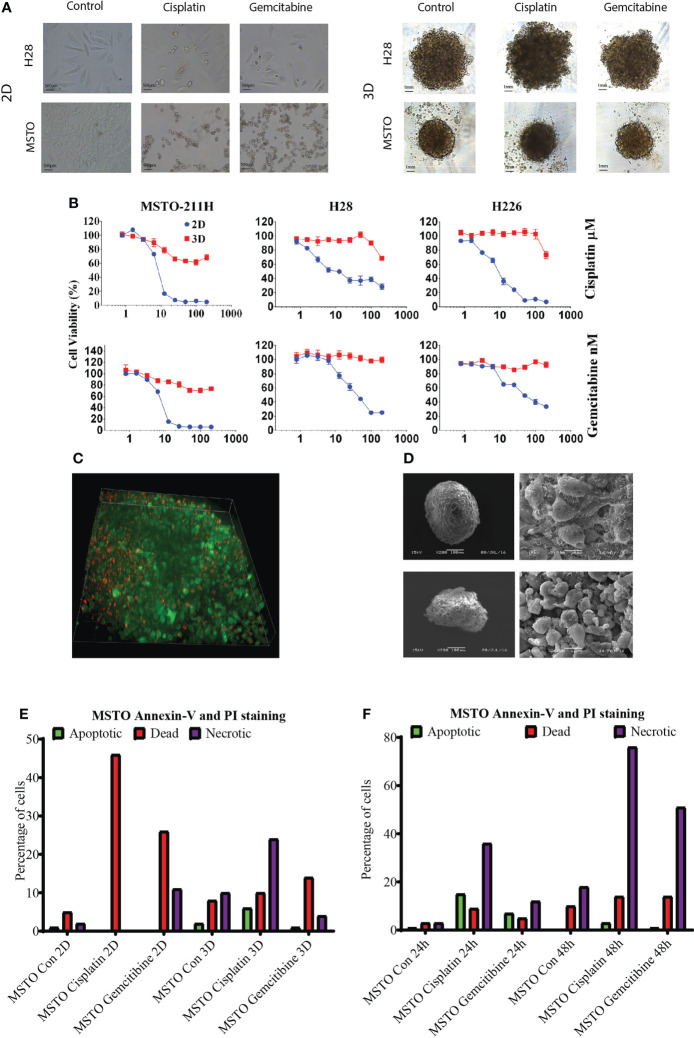
**(A)** Mesothelioma cells (MSTO, H28) were grown in 2D and LA cultured 3D spheroids and treated with cisplatin or gemcitabine for 24 hrs. **(B)** The drug response was studied by AlamaBlue proliferation assay. Mesothelioma cells (MSTO, H28, H226) grown in 3D spheroids demonstrated increased cell viability post cytotoxic drug treatment when compared to their 2D counterparts. **(C)** Drug penetration was confirmed using 3i Advanced Multimodal Microscopy. Green representing the cells and red representing Doxorubicin. **(D)** SEM analysis showed that cell junction remained tight post drug treatment in MSTO (top) and H28 (bottom) spheroids. **(E)** Cell apoptosis was analysed using a TALI image cytometer, MSTO cancer cells grown in 3D spheroids observed a reduction in dead cell number compared to MSTO cultured in 2D post drug treatment. **(F)** The number of necrotic cells was increased after 48 hrs of drug treatment compared to 24 hrs in MSTO 3D spheroids.

We then tested chemotherapy drug (cisplatin and gemcitabine) response *via* AlamarBlue proliferation assay in mesothelioma cell lines MSTO, H28 and H226. LA cultured 3D spheroids demonstrated higher resistance to cytotoxic drugs with significantly higher cell viability when compared to their 2D counterpart at the same drug concentration (**Figure 2A**, **B**, **Supplementary Table 1**). There was an average of 60% cell viability in cisplatin-treated and 80% cell viability in gemcitabine treated spheroids when compared to 20% cell viability of 2D cultured cells (**Figure 2B**). Drug penetration was studied by 3i Advanced Multimodal Microscopy to see if drug penetration influences the drug response. We demonstrated that drugs (red with Doxorubicin) well penetrated the LA cultured MSTO spheroids (**Figure 2C**; **Supplementary Video**). SEM post-drug treatment revealed that cell junctions remained tight with minimal destruction on spheroid architecture for both MSTO and H28 spheroids (**Figure 2D**). MSTO cells grown in 3D showed reduced apoptosis when compared to MSTO 2D culture, with an average of 36% fewer dead cells post cisplatin treatment, and 12% fewer dead cells post gemcitabine treatment respectively (**Figure 2E**). Meanwhile, there was an increase in the necrotic cell number in 3D cultured spheroids from 24 hrs to 48 hrs post-seeding, suggesting that drugs were slowly penetrated due to the multi-layer structure of 3D cultured spheroids (**Figure 2F**).

### MPM primary cells grown in 3D demonstrated better cell-to-cell interaction with a higher protein expression profile when compared to 2D.

**Figure 3:**
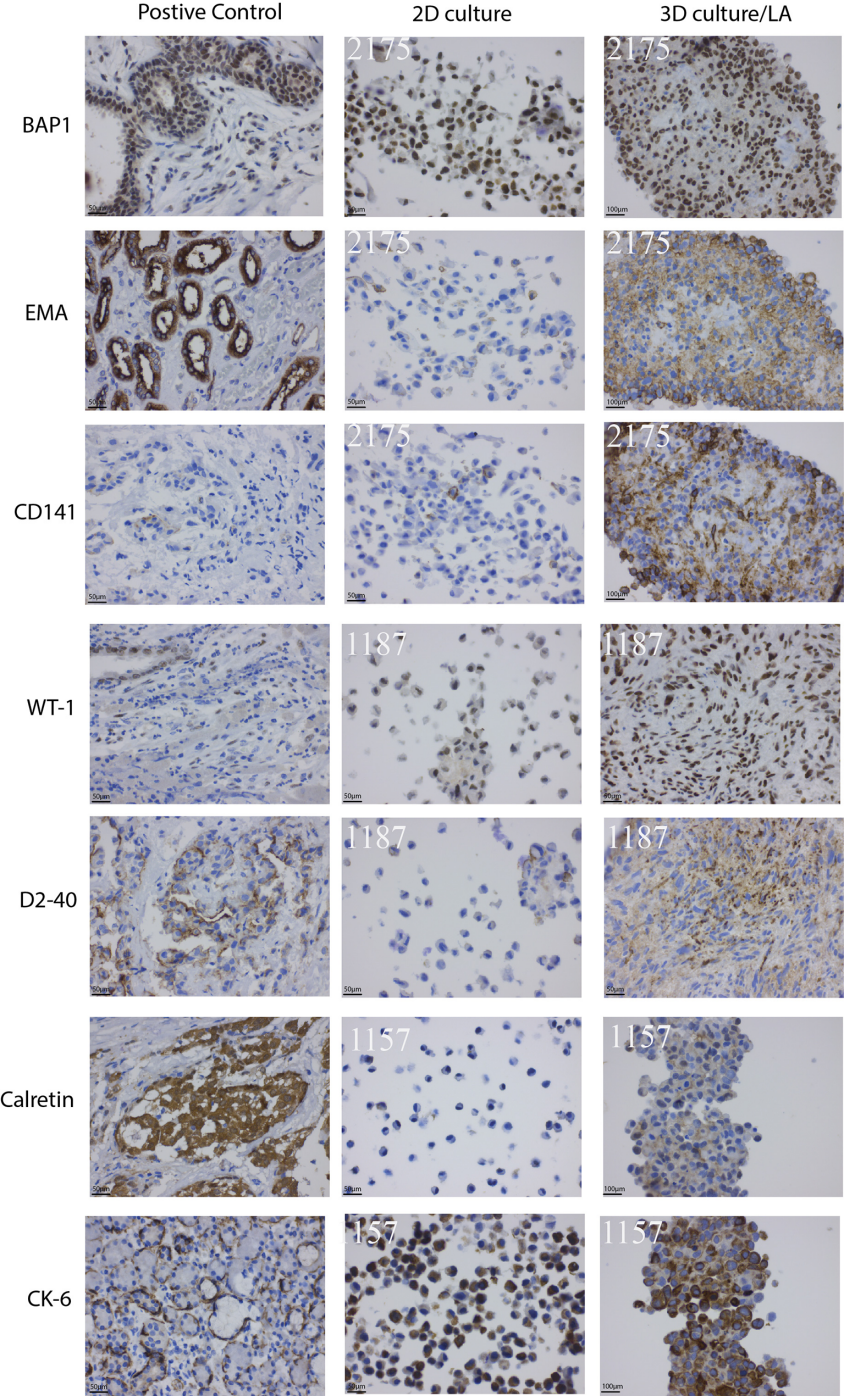
Clinically used mesothelioma biomarkers expression pattern for primary MPM cell lines. Primary MPM cell growth in 3D spheroids demonstrated an increase in IHC marker expression compared to 2D culture. Patient tissue samples were used as controls.

Nine MPM primary cell lines from the ADRI biobank were cultured using the LA spheroids. Four out of nine primary cell lines, 1170, 1180, 1137 and 1843 failed to form a spheroid structure (**Supplementary Figure 1**). 3D spheroids were then stained with H&E and showed better cell-to-cell interaction and internal architecture when compared to cells grown in 2D counterparts (**Supplementary Figure 1**). IHC staining of mesothelioma related markers including BAP1, EMA, Thrombomodulin (CD141), WT1, Podoplanin (D2-40), Calretinin and Cytokeratin6 (CK-6) revealed increased protein expression in 2175, 1187 or 1157 3D spheroids when compared to their 2D counterparts (**Figure 3**). The detailed percentage scores of protein marker expression in MPM patient cells cultured with 2D and 3D are listed in **Table 1**.

**Table 1:** Clinical used mesothelioma biomarker IHC scoring.

		BAP1	EMA	CD141	WT1	D2-40	Calretinin	CK6
2175	2D	<50%	<5%	–	–	–	–	–
	3D	60%	80%	–	–	–	–	–
1187	2D	–	–	–	80%	10%	–	–
	3D	–	–	–	90%	40%	–	–
1157	2D	–	–	–	–	–	–	70%
	3D	–	–	–	–	–	40%	90%

### MPM primary cells grown in 3D revealed different microRNA profiles expression when compared to 2D

**Figure 4:**
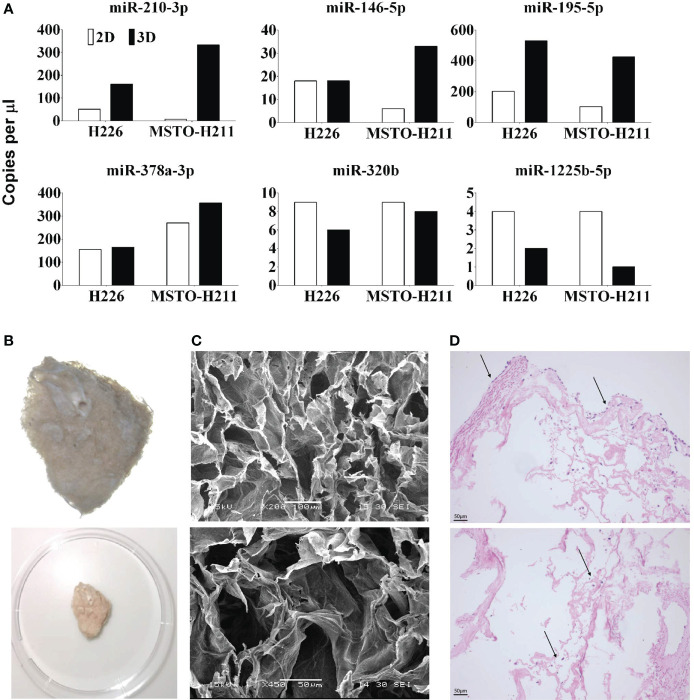
**(A)** miRNA expression qualified by dd-PCR in 2D and 3D cultured H226 and MSTO mesothelioma cells. miR 210, 146, 195 and 378 are upregulated in 3D models and miR 320, 1225 are downregulated. **(B)** Decellularized lung scaffolds are used to grow primary mesothelioma cells (26). **(C)** Physical characteristics of decellularized lung scaffolds analysed by SEM. **(D)** Mesothelioma cells can be grown in decellularized lung scaffolds from both outside (top) and inside (bottom). Up to 14 days following seeding, the mesothelioma cells are continuing to grow around the decellularized lung scaffold.

Cells grown in 3D showed different microRNA expressions when compared to their 2D counterparts. 2D and 3D datasets were analysed separately. ANOVA analysis of microRNA profiling of 2D monolayer cell lines i.e., H28, H226, MSTO, MM05 vs MeT-5A showed 14 microRNAs were significant, 12 were downregulated in MPM and 2 upregulated (hsa-miR-672 and hsa-let-7b) at P< 0.05, FDR. In 3D data, 24 miRNAs were all downregulated in MPM, P< 0.05, FDR (**Supplementary Table 2**). To investigate further gene expression changes in MPM 3D microenvironments, ANOVA analysis of 3D vs 2D dataset was carried out and 18 microRNAs were differentially expressed (P< 0.05, unadjusted). miRNA profiling revealed three microRNAs, miRNA-1255b, 15a# and 320B were down-regulated in cells grown in 3D while miR-146b, 181c,195, 210, 212, 32, 378. 500, 523 and 589 were up-regulated (**Supplementary Figure 1C**). This was further supported by ddPCR analysis which demonstrated a significant increase in the copy number of miR-210-3p, 146-5p, 195-5p and 387a-3p in 3D models when compared to 2D counterparts. The copy number of miR-1225b-5p and miR-320b in the 3D model was reduced when compared to cells grown in 2D (**Figure 4A**).

Pathway enrichment analysis on the miRDB gene targets of the 17 differentially expressed microRNAs were carried out. This is based on KEGG pathways. It is not surprising that well-known cancer pathways were enriched i.e., Wnt signaling pathway (P = 6.15E-06), MAPK signaling pathway (P = 0.0014), and PI3K-Akt signaling pathway (P = 0.003) (**Supplementary Table 3**).

To cross-examine the function of these mRNA gene targets in relation to the candidate microRNAs identified between 3D vs 2D comparison, gene enrichment analysis was carried out. Target genes that have 4 or more hits as indicated by miRDB were included (n=1147 out of 3989 genes). We noted that only 1102 has DAVID gene ID annotated (see **Supplementary Xl sheet**). Genes (n=148) involved in transcriptional activator activity, RNA polymerase II transcription regulatory region sequence-specific binding GO:0001228 were enriched (P = 1.53E-05). Other transcription related genes were enriched from the same cluster (Cluster 2). This reflected the fact that upregulated microRNAs to drive cell proliferation were activating these target genes within the regulatory cascade. Interestingly, keywords according to Uniprot https://www.uniprot.org/ for cellular component, Synapse (KW-0770) and Cell junction (KW-0965) were enriched (P = 2.68E-06, 8.50E-06 respectively). Further analysis into these gene targets (n=59 and 85 respectively) highlighted the enriched term PDZ domain, also known as discs-large homologous regions (DHR) (P = 9.0E-4 unadjusted). PDZ domain is known to be a major drug target site in cancer due to its role in signal transduction, cell–cell junctions, cell polarity and adhesion, and protein trafficking.

### A novel decellularized porcine lung scaffold grows primary mesothelioma cells over 14 days

We developed a novel scaffold model to grow primary mesothelioma cells (MM05) in 3D using the decellularized lung developed in our lab (**Figure 4B**) (26). SEM analysis of the cross-linked decellularized lung scaffold showed irregular surface and uniformed and stereoregular porous structures which are designed for cell adhesion and growth to form 3D structures (**Figure 4C**). Mesothelioma cells can be successfully grown in decellularized porcine lungs indicated with arrows, from both outside (**Figure 4D** Top) and inside (**Figure 4D** Bottom) over 14 days following seeding.

## Discussion

In the present study, we compared different methods to create 3D mesothelioma tumor models, from the simple hanging drop method to a complex novel decellularized porcine lung scaffold method. We present here for the first time comparison of strengths and weaknesses associated with a specific method. (**Table 2**).

**Table 2:** Advantages and disadvantages of the 3D cell culture methods.

3D model	Advantages	Disadvantages
Hanging drop (HD)	Simple, low cost, uniform, and controllable spheroid size	Small culture volume, the difficulty of culture medium exchange, not all lines form spheroids, therefore not suitable for every cell line
Poly-HEMA (PH)	Simple, low cost, easy to handle, suitable for high-throughput testing	Cells are not attached completely; some line does not form spheroids.
Low Adhesive (LA)	Suitable for control of spheroid (Size and parameters), relatively simple, good drug penetration and response	Difficult collecting cells for analysis, relative expensive when compared to normal culture method. Limit expansion of cell number
Porcine scaffold (PS)	Maximum resemblance to the *in vivo* condition, great drug penetration, response, and cell to cell interaction Suitable for almost every cell line.	Expensive, difficult, and time-consuming in scaffold making. Complex operation and hard to be used in large-scale production

The low adhesive (LA) cultured 3D spheroids demonstrated superior cell-to-cell junctions and smooth surface when visualized by SEM, as compared to hanging drop, poly-HEMA cultured 3D cells or their 2D counterparts. In addition, TEM revealed the unique microvilli structure that is only present in mesothelioma cells in the LA model. 3i advanced multimodal microscopy revealed cytotoxic drugs penetrated well into the spheroids. From our observation, the LA method was most reliable at producing the highest number of large spheroids needed for drug screening purposes.

However, not every primary mesothelioma tumor cell cultured by the scaffold-free models of LA, HD and PH can form spheroids (**Supplementary Figure 1**). Therefore, we utilized a decellularized porcine lung scaffold (26) that provides a superior architecture for primary mesothelioma cells to grow in, since it is a natural biomaterial making it well suited for modification to facilitate growing mesothelioma cells in 3D. The mesothelioma cells were continuing to grow around the decellularized lung scaffold both from the outside and inside up to 14 days following seeding, compared to 48hr -72hr in other 3D and 2D cultured methods. The large surface area of the alveolus bronchiole in the lung scaffolds provided more sites for initial cellular attachment and the crosslinking treatment restored the damaged collagen fibers, promoting both cell proliferation and migration (26). This decellularized porcine lung scaffold model can be used as an optimal 3D scaffold to grow patient-derived tumor cells for tumor modeling, drug development and treatment scanning due to its uniquely porous alveolus-bronchus structure having a more analogous ECM microenvironment.

We then investigated the difference in cytotoxic drug response between 2D and 3D cultured mesothelioma cells. Mesothelioma cells grown in LA spheroids demonstrated an enhanced cytotoxic drug resistance with reduced apoptosis post drug treatment when compared to their 2D counterparts. The increased cell viability in 3D spheroids can be explained by the fact that cells grow in multi-layers where interaction between tumor cells and tumor microenvironment can influence drug response. In contrast, in traditional 2D cell culture, cells grow evenly on the surface of the cell culture dish and all cells are equally exposed to a cytotoxic drug. Mesothelioma cells grown as 3D spheroids showed reduced apoptotic and necrotic rates and this may be due to both cell resistance and tissue-specific resistance mechanisms. Our findings are also supported by recent studies that demonstrated cancer cells growing in spheroids are much more resistant to chemotherapy drugs including cisplatin, paclitaxel, and doxorubicin than cells growing as a monolayer (27, 28). In past decades, numerous anti-cancer drugs were discarded during clinical trials as being ineffective indicating that anti-cancer activity tends to be overestimated in 2D-culture-based screening platforms. Cancer cells in 3D culture systems may be more appropriate as a first step in screening potential anti-cancer drugs as they better mimic the tumor microenvironment. The *in vitro* drug test system has its limitation when compared to *in vivo* models, including the difficulties to capture interaction between different cell types; problem to translate from *in vitro* drug concentration to *in vivo* dose; stimulation of long-term adverse effect when exposed to *in vivo* and the potential alteration of biomarker *in vivo*. Therefore, further studies comparing tumor formation and drug response between animal injected with mesothelioma cells cultured in 2D or 3D is required to further investigate the variability of these novel models.

Our finding suggested that the form of cellular interaction is not the factor inducing drug resistant. Whether or not the cellular interaction induced non-cellular components of the TME such as gene expression may be the key. In the present study, we detected a subset of 13 miRNA differentially expressed in mesothelioma cells cultured in spheroids compared to expression levels in cells grown in 2D. The gene expression changes were validated by dd-PCR. We observed that miRNA 210, 146, and 195 were upregulated while miRNA 320, 1225 were downregulated in multiple mesothelioma cell lines. These findings more closely recapitulate previous reports that indicate miRNA dysregulation in MPM patient tumor cells (29, 30). Pathway enrichment analysis on the miRDB gene targets of candidate microRNAs revealed that common cancer pathway including Wnt signaling pathway, MAPK signaling pathway and PI3K-Akt signaling pathway were enriched. Interestingly Axon guidance pathway is also enriched (P = 0.00057) where 48 genes (**Supplementary 2**) from this regulatory cascade can potentially be involved in tumor proliferation. In addition, genes involved in axon guidance cues and their receptors are implicated in cancer progression (31), specifically in cell migration and angiogenesis (32) (**Supplementary Table 3**).

Meanwhile, we demonstrated the increased expression of seven MPM biomarkers including BAP1, EMA, CD141, WT1, D2-40, Calretinin and CK6 in 3D cultured patient mesothelioma cancer cells compared to its 2D counterparts. These findings are consistent with our previous study of IHC expression of clinically used biomarker panels using primary MPM cell lines (21). Based on the gene-protein-function datasets described here as well as bioinformatic cross-examination of gene and pathway enrichment, we propose that 3D spheroids (LA or decellularized lung scaffold) are a better preclinical study model that best represent tumor microenvironment changes. Such system can be better utilized in biomarker discovery than 2D models.

## Conclusion

In the present study, we demonstrated technical optimization of decellularized lung scaffold to produce 3D spheroids of mesothelioma that is applicable to all mesothelioma cell types. Specifically, low-adhesive U-bottom plates is the most time effective but hanging drop and poly-HEMA methods can also result in spheroids for downstream experiments.

Gene (miRNA) profiling, protein expression and drug response of these spheroids presented here are comparable to published data. Enriched gene and pathway analyses indicated presence of genes for cell junction and cell adhesion that can only be attributed to the fact that these spheroids have cell-to-cell connections to mimic the actual tumor microenvironment. Thus, we conclude that such 3D culture system are more appropriate preclinical cancer models for drug screening and biomarker discovery.

## Data availability statement

The original contributions presented in the study are included in the article/**Supplementary Material**. Further inquiries can be directed to the corresponding author.

## Author contributions

HS and YC conceived the project, conduct the experiment and prepared the manuscript, ER and RL performed the data analysis, KHS established MM cell lines, YW performed the TEM study, and performed SEM and porcine lung scaffold preparation. KS established the porcine lung scaffold method. RL conducted gene and pathway enrichment analysis. RL, HK, BM, SoK, JM, KS, YW and StK edited the manuscript, BM collected MM sample for primary cell culture, CC and KL prepared the sections. CC, SoK and KL preformed IHC staining and analysis. All authors contributed to the article and approved the submitted version.

## Funding

This project is funded by the iCare project grant 2019-2022.

## Conflict of interest

The authors declare that the research was conducted in the absence of any commercial or financial relationships that could be constructed as a potential conflict of interest.

## Publisher’s note

All claims expressed in this article are solely those of the authors and do not necessarily represent those of their affiliated organizations, or those of the publisher, the editors and the reviewers. Any product that may be evaluated in this article, or claim that may be made by its manufacturer, is not guaranteed or endorsed by the publisher.
