# Supplementary material for: 3-Dimensional mesothelioma spheroids provide closer to natural pathophysiological tumor microenvironment for drug response studies
Source: Front Oncol. 2022 Aug 26;12:973576. doi: 10.3389/fonc.2022.973576 (PMC9462830; doi:10.3389/fonc.2022.973576)
Supplement: Supplementary file 2 [file DataSheet_1.docx]

**Supplementary Data**

**Supplementary figure 1**

Nine MPM primary cell lines cultured in 2D (A) or using the LA spheroids method (B).

(C). MPM cells grown in 3D showed difference microRNA expression when compared to their 2D counterparts.

**Supplementary table 1**

Transition points at which half the drug sensitive cells die, showing that 3D cells are more resistant to drug treatment than 2D. The IC50 transition point at which half of all cells die was reached for 2D cell types. For 3D cell types, the IC50 transition point was either higher than that of the corresponding 2D cell type or was not reached at all which signifies that it was higher than the highest dose tested. Values not applicable are displayed with a dash ‘–’.

| **Cell line** | **Cell type** | **Drug** | **Primary transition point (IC50)** | **Percentage of viable cells after primary transition point (IC50)** | **Secondary transition point** | **Percentage of viable cells after secondary transition point** |
| --- | --- | --- | --- | --- | --- | --- |
| MSTO-211 | 2D | Cisplatin | 2.42 µm | 6% | – | – |
| MSTO-211 | 3D | Cisplatin | not reached | – | 2.68 µm | 64% |
| H28 | 2D | Cisplatin | 1.41 µm | 31% | – | – |
| H28 | 3D | Cisplatin | 10.76 µm | 20% | – | – |
| H226 | 2D | Cisplatin | 2.53 µm | 20% | – | – |
| H226 | 3D | Cisplatin | 10.43 µm | 20% | – | – |
| MSTO-211 | 2D | Gemcitabine | 2.39 nm | 6% | – | – |
| MSTO-211 | 3D | Gemcitabine | not reached | – | 2.14 nm | 69% |
| H28 | 2D | Gemcitabine | 3.95 nm | 20% | – | – |
| H28 | 3D | Gemcitabine | not reached | – | 9.14 nm | 90% |
| H226 | 2D | Gemcitabine | 3.87 nm | 29% | – | – |
| H226 | 3D | Gemcitabine | not reached | – | – | – |

**Supplementary table 2**

ANOVA analysis of microRNA profiling of 2D cell lines i.e., H28, H226, MSTO, MM05 vs MeT-5A showed 14 microRNAs were significant, 12 were downregulated in MPM and 2 upregulated (has-miR-672 and has-let-7b) at P < 0.05, FDR. In 3D data, 24 miRNAs were all downregulated in MPM, P< 0.05, FDR.

The following microRNA nomenclatures were curated i.e., 3D: 92b* = hsa-miR-92b-5p; 181c* = 181c-3p; 125b-1* = 125b-1-3p; 132* = 132-5p; 143* = 143-5p; and 2D: 145* = 145-3p; 23a* = 23a-5p;    186* = 186-3p; let-7f-1* = 7f-1-3p; 9* = 9-3p.

| **2D** | **microRNA** | **p-value**  **(MPM vs. Control)** | **Fold-Change**  **(MPM vs. Control)** | **Fold-Change**  **(MPM vs. Control) (Description)** |
| --- | --- | --- | --- | --- |
|  | **hsa-miR-615-5p** | 0.000144486 | -536.617 | MPM down vs Control |
|  | **hsa-miR-34b-3p** | 0.000430223 | -78.2354 | MPM down vs Control |
|  | **hsa-miR-145-3p** | 0.00043063 | -78.127 | MPM down vs Control |
|  | **hsa-miR-23a-5p** | 0.000669268 | -42.8359 | MPM down vs Control |
|  | **hsa-miR-570-3p** | 0.000733956 | -38.1801 | MPM down vs Control |
|  | **hsa-miR-186-3p** | 0.000819094 | -33.4457 | MPM down vs Control |
|  | **hsa-miR-1278** | 0.00105564 | -25.0676 | MPM down vs Control |
|  | **hsa-miR-885-3p** | 0.00123792 | -21.1671 | MPM down vs Control |
|  | **hsa-miR-369-5p** | 0.00139712 | -18.7231 | MPM down vs Control |
|  | **hsa-let-7f-1-3p** | 0.00156075 | -16.8042 | MPM down vs Control |
|  | **hsa-miR-9-3p** | 0.0016221 | -16.1981 | MPM down vs Control |
|  | **hsa-miR-486-5p** | 0.00177067 | -14.9259 | MPM down vs Control |
|  | **hsa-let-7b-3p** | 0.00115118 | 1.58804 | MPM up vs Control |
|  | **mmu-miR-672-5p** | 0.00164014 | 5.44243 | MPM up vs Control |

| **3D** | **microRNA** | **p-value(MPM vs. Control)** | **Fold-Change(MPM vs. Control)** | **Fold-Change(MPM vs. Control) (Description)** |
| --- | --- | --- | --- | --- |
|  | **hsa-miR-1238-3p** | 1.68E-05 | -2120.96 | MPM down vs Control |
|  | **mmu-miR-499-5p** | 6.14E-05 | -144.057 | MPM down vs Control |
|  | **mmu-miR-674-5p** | 7.10E-05 | -113.811 | MPM down vs Control |
|  | **hsa-miR-92b-5p** | 7.53E-05 | -103.86 | MPM down vs Control |
|  | **hsa-miR-615-5p** | 8.13E-05 | -92.3794 | MPM down vs Control |
|  | **hsa-miR-548d-3p** | 9.62E-05 | -72.1286 | MPM down vs Control |
|  | **hsa-miR-369-5p** | 0.000112934 | -57.66 | MPM down vs Control |
|  | **hsa-miR-181c-3p** | 0.000132899 | -46.511 | MPM down vs Control |
|  | **hsa-miR-34b-3p** | 0.000195573 | -29.192 | MPM down vs Control |
|  | **hsa-miR-544a** | 0.000207946 | -27.256 | MPM down vs Control |
|  | **hsa-miR-1276** | 0.000216367 | -26.0914 | MPM down vs Control |
|  | **hsa-miR-132-5p** | 0.000256703 | -21.7584 | MPM down vs Control |
|  | **hsa-miR-1294** | 0.000275112 | -20.2732 | MPM down vs Control |
|  | **hsa-miR-125b-1-3p** | 0.000386771 | -14.6467 | MPM down vs Control |
|  | **hsa-miR-296-3p** | 0.000416125 | -13.7228 | MPM down vs Control |
|  | **hsa-miR-657** | 0.000438177 | -13.1182 | MPM down vs Control |
|  | **hsa-miR-377-3p** | 0.000618739 | -9.89363 | MPM down vs Control |
|  | **hsa-miR-373-3p** | 0.000813174 | -8.08641 | MPM down vs Control |
|  | **hsa-miR-143-5p** | 0.00111119 | -6.55911 | MPM down vs Control |
|  | **hsa-miR-342-5p** | 0.00147077 | -5.53085 | MPM down vs Control |
|  | **hsa-miR-640** | 0.00153582 | -5.39454 | MPM down vs Control |
|  | **hsa-miR-576-5p** | 0.00194304 | -4.73873 | MPM down vs Control |
|  | **hsa-miR-1197** | 0.00221167 | -4.43059 | MPM down vs Control |
|  | **hsa-miR-345-5p** | 0.00277147 | -38.459 | MPM down vs Control |

**Supplementary table 3.**

Pathway enrichment analysis based on KEGG (https://www.genome.jp/kegg/) on the gene targets of the 17 differentially expressed miRNA.

| **Pathway Name** | **Database** | **Enrichment Score** | **Enrichment p-value** | **# genes in list, in pathway** |
| --- | --- | --- | --- | --- |
| Wnt signaling pathway | KEGG | 11.9985 | 6.15E-06 | 49 |
| Glycerophospholipid metabolism | KEGG | 7.48497 | 0.000561462 | 33 |
| Axon guidance | KEGG | 7.4631 | 0.000573875 | 48 |
| Melanogenesis | KEGG | 7.00833 | 0.000904314 | 35 |
| Ubiquitin mediated proteolysis | KEGG | 7.00575 | 0.000906655 | 48 |
| Proteoglycans in cancer | KEGG | 6.97516 | 0.000934819 | 79 |
| FoxO signaling pathway | KEGG | 6.85714 | 0.00105192 | 53 |
| Hippo signaling pathway | KEGG | 6.55337 | 0.00142531 | 50 |
| MAPK signaling pathway | KEGG | 6.50337 | 0.00149838 | 90 |
| Morphine addiction | KEGG | 6.2241 | 0.0019811 | 26 |
| Phosphatidylinositol signaling system | KEGG | 5.97395 | 0.00254418 | 27 |
| PI3K-Akt signaling pathway | KEGG | 5.63664 | 0.00356484 | 99 |
| Hedgehog signaling pathway | KEGG | 5.46103 | 0.00424918 | 17 |
| Ether lipid metabolism | KEGG | 5.25579 | 0.00521723 | 16 |
| Circadian rhythm | KEGG | 5.18698 | 0.00558885 | 14 |
| Transcriptional misregulation in cancer | KEGG | 4.9941 | 0.0067778 | 58 |
| Pathways in cancer | KEGG | 4.98113 | 0.00686629 | 104 |
| Neurotrophin signaling pathway | KEGG | 4.97044 | 0.00694008 | 45 |
| Gastric acid secretion | KEGG | 4.90086 | 0.00744017 | 24 |
| Renal cell carcinoma | KEGG | 4.85253 | 0.00780858 | 28 |
| Dopaminergic synapse | KEGG | 4.70236 | 0.00907385 | 41 |
| Oocyte meiosis | KEGG | 4.56901 | 0.0103682 | 34 |
| Dorso-ventral axis formation | KEGG | 4.42784 | 0.0119403 | 12 |
| Insulin signaling pathway | KEGG | 4.21573 | 0.0147616 | 43 |
| Glutamatergic synapse | KEGG | 4.18553 | 0.0152142 | 34 |
| Focal adhesion | KEGG | 3.96392 | 0.0189885 | 59 |
| p53 signaling pathway | KEGG | 3.92093 | 0.0198226 | 23 |
| Ras signaling pathway | KEGG | 3.89652 | 0.0203124 | 64 |
| Hepatitis B | KEGG | 3.81427 | 0.0220537 | 49 |
| Inositol phosphate metabolism | KEGG | 3.80478 | 0.0222641 | 19 |
| Retrograde endocannabinoid signaling | KEGG | 3.78962 | 0.0226041 | 30 |
| mTOR signaling pathway | KEGG | 3.78237 | 0.0227686 | 24 |
| Cell cycle | KEGG | 3.72006 | 0.0242325 | 37 |
| Basal cell carcinoma | KEGG | 3.71611 | 0.0243285 | 16 |
| Glycosaminoglycan biosynthesis - keratan sulfate | KEGG | 3.58731 | 0.0276728 | 8 |
| Endocytosis | KEGG | 3.42834 | 0.0324407 | 57 |
| Fc gamma R-mediated phagocytosis | KEGG | 3.34906 | 0.0351174 | 29 |
| Protein processing in endoplasmic reticulum | KEGG | 3.33638 | 0.0355656 | 49 |
| Sphingolipid metabolism | KEGG | 3.32595 | 0.0359384 | 15 |
| Pancreatic secretion | KEGG | 3.32316 | 0.0360388 | 27 |
| Selenocompound metabolism | KEGG | 3.29648 | 0.0370133 | 7 |
| GABAergic synapse | KEGG | 3.28876 | 0.0373 | 23 |
| Mucin type O-Glycan biosynthesis | KEGG | 3.07876 | 0.0460162 | 11 |
| Cocaine addiction | KEGG | 3.07593 | 0.0461469 | 16 |
| Protein export | KEGG | 3.07022 | 0.0464111 | 7 |
| Cholinergic synapse | KEGG | 3.05512 | 0.047117 | 33 |
| Salivary secretion | KEGG | 3.03422 | 0.0481121 | 24 |
