# Supplementary figures and images for: 3-Dimensional mesothelioma spheroids provide closer to natural pathophysiological tumor microenvironment for drug response studies
Source: Front Oncol. 2022 Aug 26;12:973576. doi: 10.3389/fonc.2022.973576 (PMC9462830; doi:10.3389/fonc.2022.973576)

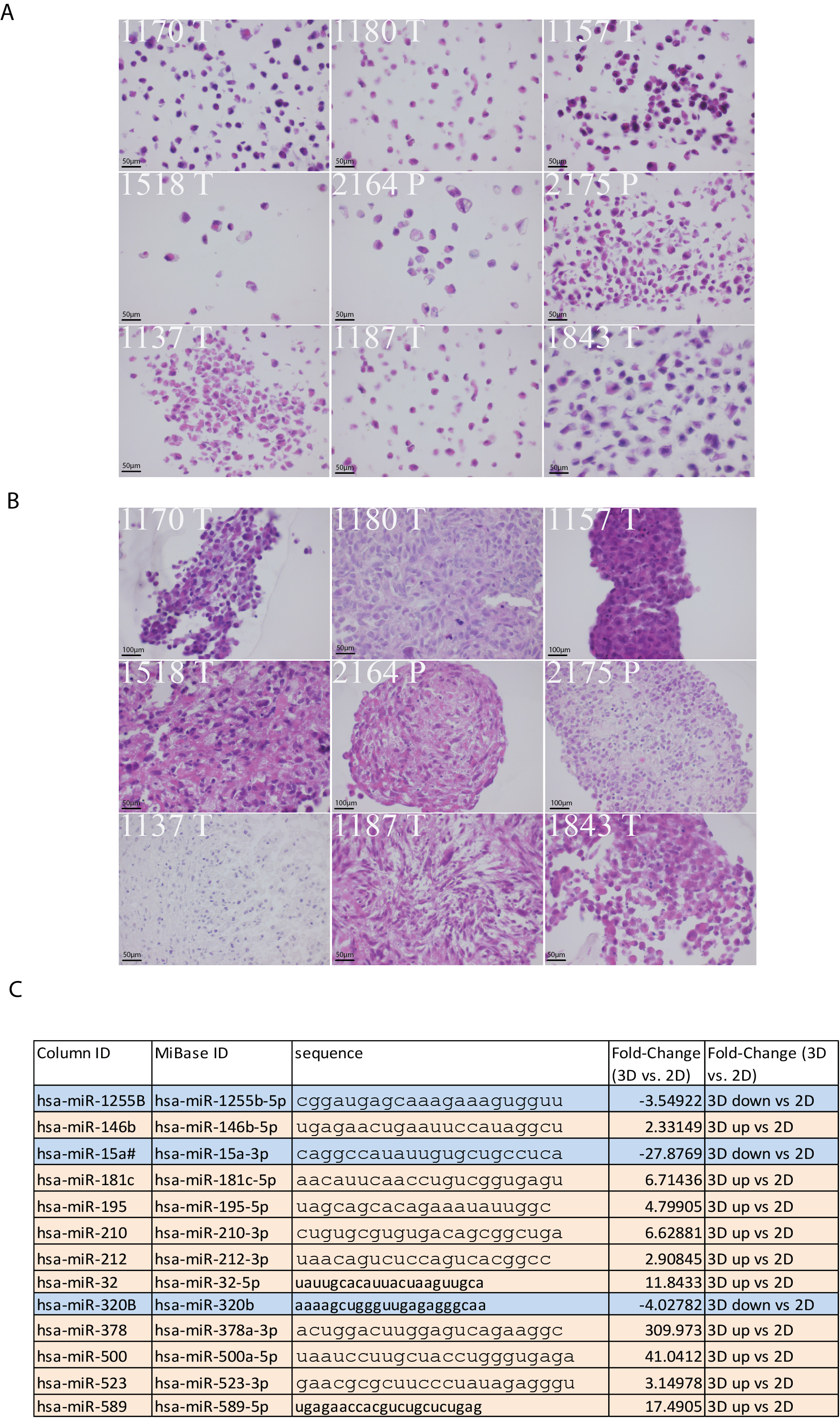

Supplement: Supplementary file 4 [file Image_1.jpeg]
